# Diffusion Tensor Imaging Study of Olfactory Identification Deficit in Patients With Mild Cognitive Impairment

**DOI:** 10.3389/fnagi.2021.765432

**Published:** 2021-11-23

**Authors:** Yongjia Shao, Zijian Wang, Bin Ji, Hang Qi, Shangci Hao, Gang Li, Yue Zhang, Qian Xi

**Affiliations:** ^1^Department of Radiology, Shanghai East Hospital, Tongji University School of Medicine, Shanghai, China; ^2^School of Computer Science and Technology, Donghua University, Shanghai, China; ^3^Department of Neurology, Shanghai East Hospital, Tongji University School of Medicine, Shanghai, China

**Keywords:** diffusion tensor imaging, tract-based spatial statistics, mild cognitive impairment, Alzheimer’s disease, olfactory dysfunction

## Abstract

**Objective:** To explore the relationship between white matter changes and olfactory ability among patients with mild cognitive impairment (MCI) and to develop a tool to predict the development of Alzheimer’s disease among patients with MCI.

**Methods:** The Montreal Cognitive Assessment (MoCA) was used for cognitive assessments, and the 70% isopropanol test paper was used to evaluate olfactory function. Tract-based spatial statistics, based on the diffusion tensor imaging technology, were used to obtain relevant parameters, and behavioral and imaging results were compared between patients with MCI (*n* = 36) and healthy older adults (*n* = 32).

**Results:** The olfactory ability of MCI patients was lower overall, which was positively correlated with the MoCA score. Fractional anisotropy (FA) changes significantly of all parameters. Lower FA regions were mainly located in the corpus callosum, the orbitofrontal gyrus, and the left occipital lobe. The olfactory score was significantly correlated with the FA value of the orbitofrontal gyrus. Fibrous connections in several brain regions, such as the entorhinal cortex, were stronger in patients with MCI.

**Conclusion:** The olfactory ability of MCI patients in our group was positively correlated with the neuropsychological scale results. Impairment in olfactory function was superior to memory deficits for predicting cognitive decline among cognitively intact participants. The fibrous connections in several brain regions, such as the entorhinal cortex, were higher in patients with MCI, which suggested that there may be a compensatory mechanism in the olfactory pathway in MCI patients. The decline in olfactory function may be a significant and useful indicator of neuropathological changes in MCI patients and an effective marker for the development of cognitive decline and dementia.

## Introduction

Alzheimer’s disease (AD), which is characterized by latent and progressive cognitive decline, is the most common etiology for dementia and is recognized as an urgent concern that has significant implications for both individuals and society (Lane et al., 2018). Mild cognitive impairment (MCI), which is considered a transitional state between normal aging and dementia, has a conversion range of 8–15% per year (Shu et al., 2021). In line with recommendations form the National Institute on Aging-Alzheimers’s Association workgroups, we use the term “MCI due to AD” to refer to the symptomatic predementia phase of AD, which suggests that MCI is a critical stage for preventive treatment for dementia (Albert et al., 2011). Therefore, there is significant interest in developing a sensitive, specific, and non-invasive method for the early prediction of MCI before progression to AD. At present, neuropsychological tests and neuroimaging technology, such as positron emission tomography (PET), cerebrospinal fluid (CSF) measurements, and magnetic resonance imaging (MRI), are commonly used for early diagnosis of MCI (Jung et al., 2019). However, neuropsychological tests are subjective and are easily affected by factors, such as subject’s mood, mental status, and educational background. Furthermore, the high cost of PET and the invasive nature of obtaining CSF limit their utility. Neuroimaging methods have proliferated in recent years; among them, diffusion-tensor imaging (DTI) is a non-invasive neuroimaging modality used to evaluate the structure of white matter in the brain, which is currently the only way to map white matter fiber architecture in brain tissue. Fractional anisotropy (FA), mean diffusivity (MD), radial diffusivity (DR), and axial diffusivity (DA; Georgiopoulos et al., 2017) are the most commonly used DTI metrics. Among these, FA and MD are most frequently reported. In DTI, the displacement of water molecules is used to measure white matter tract integrity. FA assesses the degree of directionality of water diffusion, whereas MD measures the mean water diffusion rate.

The olfactory center of the brain is composed of the primary (POC) and secondary olfactory cortices (SOC). The POC plays an important role in olfactory detection and has a complex structure that mainly comprises the entorhinal cortex (EC), amygdala, and piriform cortex (Kjelvik et al., 2020). The SOC is involved in olfactory recognition and mainly includes the hippocampus, hypothalamus, orbitofrontal gyrus, striatum, and corpus callosum. Among them, the orbitofrontal gyrus is the highest olfactory center, that determines the pleasure and familiarity of odors (Zhao et al., 2016). The olfactory pathway not only overlaps with hippocampus, the typical lesion site of AD, but also is involved in early pathological changes of AD (Marin et al., 2018). In line with a recent study, AD-related pathological alterations first resulted in synaptic neurodegeneration and then neuronal loss, it found no significant neuronal loss in the EC was detectable in cognitively normal subjects, by contrast, there was a very severe neuronal loss in the EC even in very mild AD cases (Li et al., 2021). The results highlighted the EC maybe helpful for predicting the pre-symptomatic and very mild stages of AD, therefore, olfactory function assessment provides the possibility for early recognition of cognitive dysfunction. Numerous recent studies applied neuroimaging modality to identify a clinical marker for predicting the preclinical AD, a study verified a strong correlation between olfactory impairment and white matter damage (Woodward et al., 2017). Some have also found, compared with cognitively normal controls (NC), patients with MCI were shown to have lower FA and higher MD in the hippocampus, EC, medial temporal lobe, and corpus callosum (Knight et al., 2019), which overlap with several of the olfactory functional areas described above. In addition, one study reported patients with more severe cognitive dysfunction had worse olfactory function (Yoo et al., 2018). All of these findings suggest that olfactory identification (OI) is a significant factor for predicting the risk of MCI-to-AD transition (Devanand, 2016). However, further research is needed to identify a visual indicator for predicting the development of AD in MCI patients with OI impairment.

In this prospective study, DTI and tract-based-spatial-statistics (TBSS) were used to conduct a cross-sectional analysis of white matter microstructural changes in MCI patients and NC to explore the relationship between white matter changes and olfactory ability in patients with MCI and to develop a reliable method for improving diagnoses and reducing underdiagnoses of MCI and dementia.

## Materials and Methods

### Study Participants

Sixty-eight subjects (36 MCI and 32 NC) were recruited from the Shanghai Jinguang community from March 2017 to December 2019. All patients were recruited by the Neurology Department of Shanghai East Hospital (Southern Branch) after they underwent neuropsychological tests. The study was reviewed and approved by the Ethics Review Committee of Shanghai East Hospital, and written informed consent was obtained from all participants.

The Mini-Mental State Examination (MMSE) is the most widely used screening scale owing to its speed and ease of administration. However, its sensitivity in identifying patients with MCI, AD and healthy people is relatively low (Albert et al., 2011). While, the Montreal Cognitive Assessment (MoCA) covers a wider range of cognitive domains, including attention and concentration, executive function, memory, language, visual-spatial structure skills, abstract thinking, computation and orientation, therefore, it has superior sensitivity and specificity to the MMSE in predicting cognitive decline (Lu et al., 2011).

Inclusion criteria for MCI patients were the following: (1) a subjective complaint of mild cognitive decline by the patient, preferably confirmed by an insider; (2) minimal effect of working and living independently and handling complex tasks; (3) objective evidence of memory loss, with an MMSE score ≤27 and ≥24; (4) absence of dementia, with a MoCA score ≤24 (illiterate group ≤13 points, primary school group ≤19 points, middle school and above group ≤24 points), based on MoCA for detecting MCI in Chinese older adults (Lu et al., 2011).

Inclusion criteria for NC were the following: (1) independent behavioral ability, no cognitive or memory impairment, no depression, and no organic nervous system diseases; and (2) a MoCA score >24 (Lu et al., 2011).

Exclusion criteria were the following: (1) history of nervous system tumor, craniocerebral surgery, stroke, or brain trauma; (2) serious medical diseases, such as organ dysfunction, autoimmune diseases, blood system diseases, anemia, and tumor; (3) unable to cooperate with examination because of a mental disorder, speech confusion, or severe hearing impairment; and (4) other neurodegenerative diseases causing cognitive impairment, such as bipolar disorder, mania, schizophrenia, epilepsy, PD, and multiple sclerosis.

### Olfactory Identification Test

The most commonly used odor identification test is the University of Pennsylvania Smell Identification Test (UPSIT), however, it takes too long to administer by using 40 scents. Furthermore, too much odor identification can be affected by differences in region, culture, and individual experiences, so it is complicated to use as a screening test for the elderly (Kim et al., 2020, 2021). The “Sniffin’ Sticks” Test (SST) is another widely used odor test, but its applicability is limited because of the high cost and the regional-cultural differentiation of odor recognition (Demir et al., 2021). Therefore, all of our subjects underwent the Alcohol Sniff Test, which is a simple, rapid, and reliable measure of olfaction, it takes less than 5 min to administer, has good test-retest reliability and can be used cross-culturally, which is easy to use in the elderly (Davidson et al., 1998). In a quiet, ventilated, and private environment, subjects kept their eyes and mouth closed while the researcher placed a soft ruler under the subject’s nose with the proximal end of the ruler perpendicular to the tip of the nose. The isopropyl alcohol (70 g/100 ml) test paper was placed under the subject’s nose at the 30 cm mark of the soft ruler. The subject was then instructed to take a deep breath while the researcher simultaneously moved the test paper in 1 cm increments up the ruler with each breath until the subject could smell an isopropyl alcohol odor. The test was repeated four times, and the average distance was calculated. The score was based on the distance from the tip of the nose to the test paper, which indicated anosmia (average distance <10 cm), hyposmia (10 cm ≤average distance ≤15 cm), or normal smell (average distance >15 cm) (Ashwin et al., 2014).

### Magnetic Resonance Imaging

All participants underwent a DTI scan on an M750w 3.0T GE Signa MRI system (GE Healthcare, American) with a 32-channel head and neck coil. During scanning, all subjects lay in a supine position with their head positioned in the center of the coil, and earplugs were placed in their ears to reduce scanner noise. Participants were asked to minimize head movements as much as possible. T1-weighted three-dimensional (3D-T1), axial T2-weighted, and fluid-attenuated inversion recovery images constituted the structural imaging, which was used to exclude abnormalities other than atrophy or white matter degeneration. 3D-T1 images were acquired using a fast-spoiled gradient recalled echo sequence [repetition time (TR) = 8.5 ms, echo time [TE] = 3.2 ms, field of view [FOV] = 256 × 256 mm, slice thickness = 1 mm]. In addition, diffusion-weighted imaging (DWI) and DTI were acquired simultaneously. The DWI sequence parameters were: TR = 13700 ms, TE = 85 ms, FOV = 224 × 224 mm, slice thickness = 2 mm. The DTI sequence parameters were: TR = 13701 ms, TE = 114 ms, FOV = 224 × 224 mm, slice thickness = 2 mm, diffusion coefficient b = 1000 s/mm^2^, 64 diffusion-sensitive gradients, one *b* = 0 s/mm^2^, and 70 continuous slices in each gradient direction.

### Imaging Processing

3D-T1 images were reviewed by two experienced radiologists to check for any morphological abnormalities. The regions of interest (ROIs) were determined jointly by neurologists and radiologists who had no knowledge of patient information, including the hippocampus, the corpus callosum, the orbitofrontal gyrus and the left inferior occipital gyrus. Post-processing of DTI data was performed using the Functional Magnetic Resonance Imaging of the Brain (FMRIB) Software Library version 5.0^1^, which contains TBSS and FMRIB’s diffusion toolbox (FDT). Original DTI images were corrected for head movement and eddy current distortions, which was followed by brain extraction to eliminate non-brain tissue and brain mask generation to ensure inclusion of the ventral surface of the forebrain using the Brain Extraction Tool (BET). Subsequently, commonly used DTI metrics were estimated, derived and calculated using FDT, such as FA, MD, DA, and DR, which applies a diffusion tensor model to describe fibrous structural characteristics that indicate white matter microdamage (Tae et al., 2018). Then, we used the abnormal area based on the whole-brain TBSS analysis results as the seed region to perform probabilistic diffusion tractography with a probability of over 90%, and the specific process mainly includes tracer modeling, image registration and tracer analysis.

### Statistical Analysis

Statistical calculations were performed with the Statistical Package for the Social Sciences version 23.0 IBM. Means ± standard deviations (SD) were used to express measurement data, which were assessed for normality test prior to statistical analysis, and then the two independent sample *t*-tests were carried out according to the normality test result; whereas chi-squared tests were used for enumeration data. The FSL-Randomize function was used to extract the average values of FA, DA, DR, and MD of the whole brain in the experimental and control group, and then statistical tests were performed on the average values of above DTI metrics, and inter- and intra-group statistical analyses of DTI data for ROIs were performed. In addition, correlation analyses were conducted between the behavioral indicators and olfactory test values, as well as between the behavioral and the imaging indicators. At the same time, we make the receiver operating characteristic (ROC) curve to reveal the correlation between sensitivity and specificity.

## Results

### Clinical Characteristics of the Study Cohort

Results showed that there were no significant differences in sex ratio, age, or educational background between the groups (*p* > 0.05, Table 1). However, OI and MoCA scores were significantly different between the experimental and control groups (*p* < 0.05, Table 1).

**TABLE 1:** Demographic information of participants.

**Group**	**MCI (*n* = 36)**		**NC (*n* = 32)**		**Test**	
	**Mean**	**SD**	**Mean**	**SD**	***t***	***p***
Age (year)	67.794	6.3331	68.810	5.5463	−0.6050	0.548
Education background (year)	9.2060	2.4093	9.1430	2.0071	0.1000	0.921
OI score	10.118	3.8672	17.143	2.3084	−7.5220	<0.001
MoCA score	17.412	6.2091	27.000	1.4491	−6.9380	<0.001

	**N**	**Female (%)**	**N**	**Female (%)**	***F***	***X* ^2^**

Sex ratio	36.000	58.300	32.000	42.900	1.0000	0.1710

### Correlational Analysis of Behavioral Indicators

**FIGURE 1:**
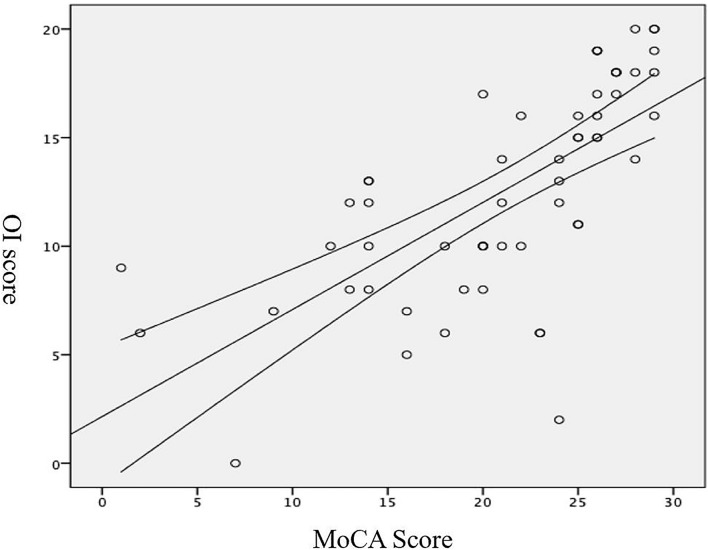
Correlation between the MoCA and OI scores.

**FIGURE 2:**
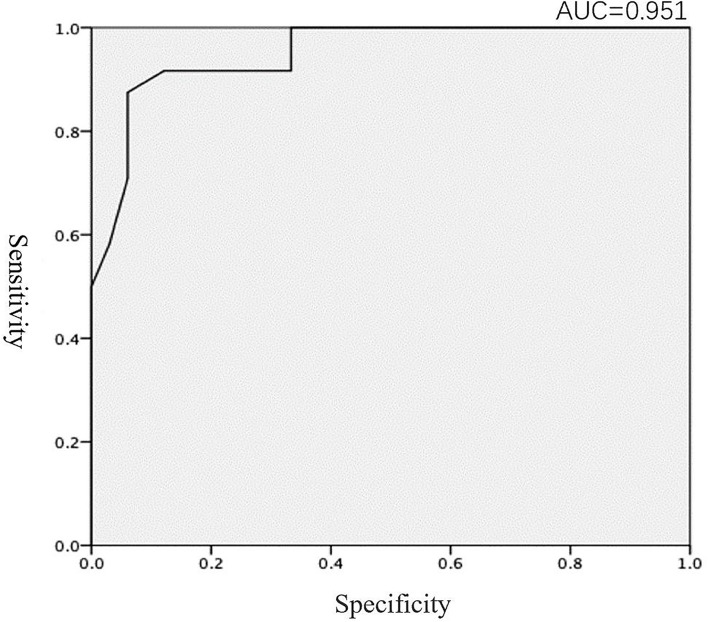
ROC curve of the MoCA and OI scores.

Patients with MCI had significantly poorer performance on the OI test than the NC, and there were significant differences in the OI and MoCA scores between the MCI and NC groups (*p* < 0.01, Table 2). After MoCA score was converted into ranked information, the Pearson correlation test showed that the olfactory function of patients with MCI was poorer than NC, and neuropsychological test results were positively correlated with the olfactory test value (*r* = 0.682, *p* < 0.01; Table 3 and Figure 1). In addition, we found that the value of the olfaction test had high specificity for predicting MCI (area under the curve = 0.951; Figure 2).

**TABLE 2:** Comparison of behavioral score between MCI patients and NCs.

	**MCI (*n* = 36)**	**NC (*n* = 32)**	***P***
OI score	10.12 ± 3.87	17.14 ± 2.31	<0.010
MoCA score	17.41 ± 6.21	27.00 ± 1.45	<0.010

**TABLE 3:** Correlation between MoCA score and OI score.

		**MoCA score**	**OI score**
MoCA score	*r*	1.000	0.682
	*p*	–	<0.001
OI score	*r*	0.682	1.000
	*p*	<0.001	–

### Correlational Analysis of Imaging Indicators

**FIGURE 3:**
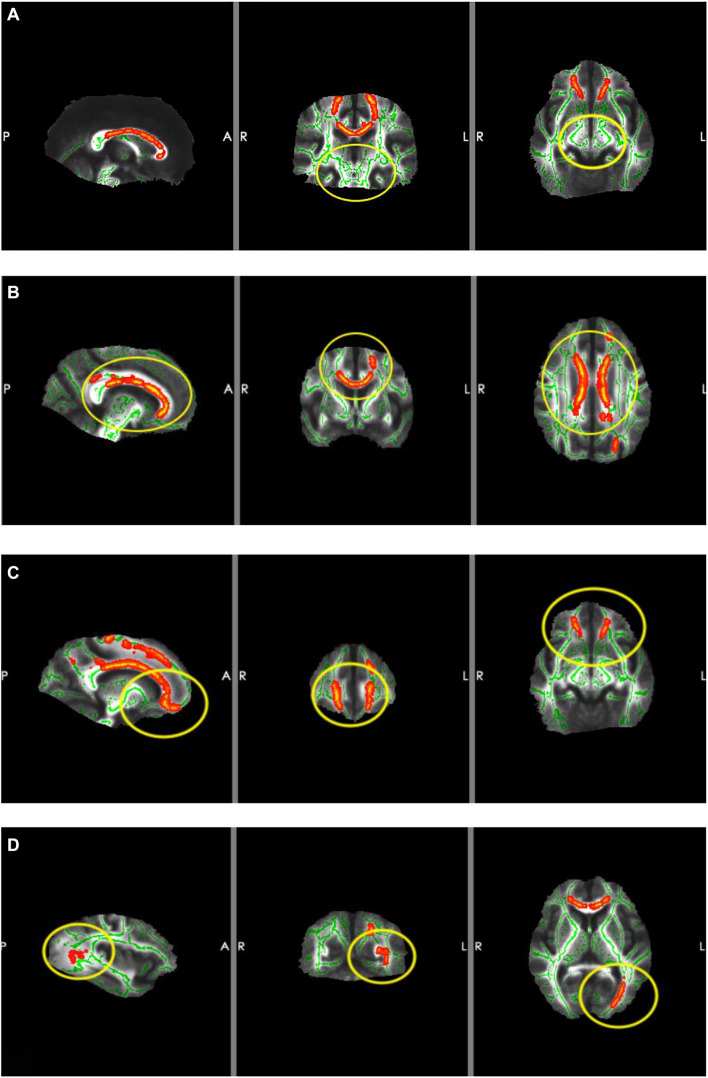
Results of TBSS. The red areas are the areas of lower FA (active area) and the yellow circles are the ROIs. **(A)** ROI located around the hippocampus (there were no significant changes in FA). **(B)** ROI located around the corpus callosum (FA was lower in MCI patients). **(C)** ROI located around the orbitofrontal gyrus (FA was lower in MCI patients). **(D)** ROI located around the left inferior occipital gyrus (FA was lower in MCI patients).

Compared with the NC group, the MCI group showed significantly higher DR and lower FA (*p* < 0.05; Table 4). Whole-brain TBSS analysis showed that the areas of lower FA (active area) were primarily localized to areas around the corpus callosum, orbitofrontal gyrus, and left inferior occipital gyrus. However, there was no significant FA difference around the hippocampus (Figure 3).

**TABLE 4:** Whole-brain DTI analyses.

	**MCI**	**NC**	***t***	***p***
FA	0.67200 ± 0.02800	0.69100 ± 0.01600	−2.814	0.007
MD	0.00069 ± 0.00003	0.00068 ± 0.00002	1.1230	0.268
DA	0.00130 ± 0.00003	0.00130 ± 0.00004	−1.139	0.261
DR	0.00037 ± 0.00004	0.00035 ± 0.00002	2.1110	0.040

**FIGURE 4:**
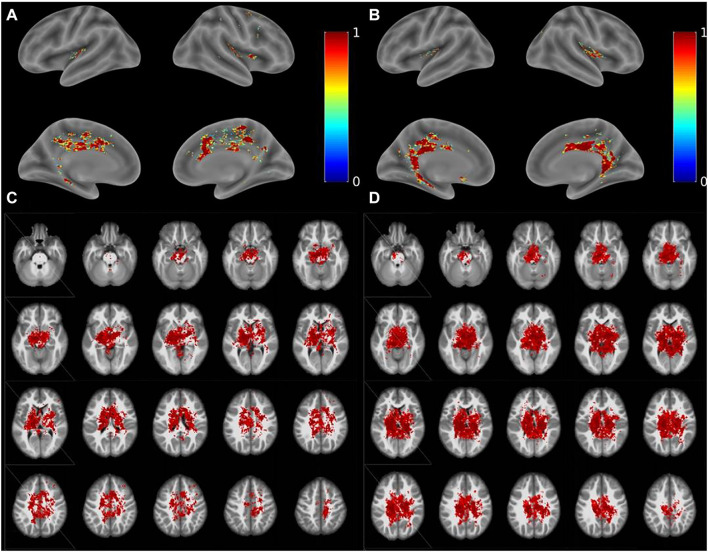
Results of diffusion tractography. **(A,C)** Diffusion tractography results of the MCI group. **(B,D)** Diffusion tractography results of the NC group.

**FIGURE 5:**
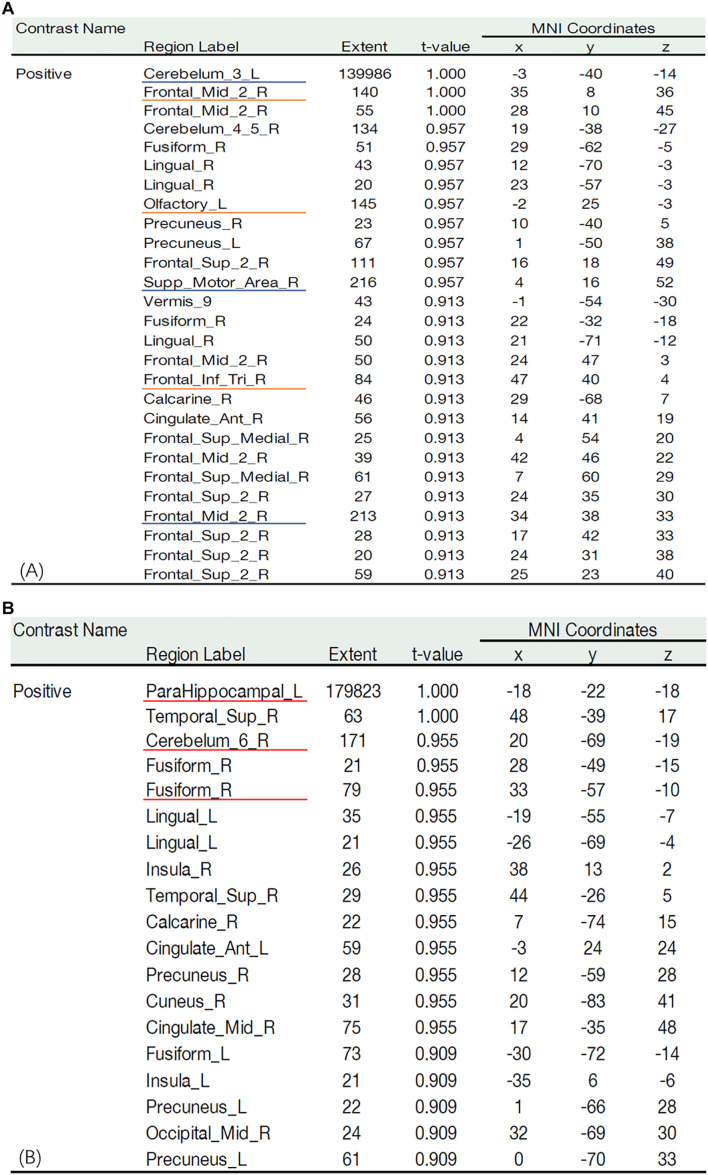
Statistic map of the fiber probability tracking. **(A)** Statistic map of the fiber probability tracking in the MCI group. Blue underline is on behalf of the areas which a large number of fibers mainly reached in the MCI group. Orange underline is on behalf of some areas which can be observed in the MCI group, but can’t be observed in the NC group. **(B)** Statistic map of the fiber probability tracking in the NCI group. Red underline is on behalf of the areas which a large number of fibers mainly reached in the NC group.

Compared with the NC group, the fiber tracking connection area (red area) in the MCI group was significantly lower (Figure 4). In the MCI group, a large number of fibers reached the cerebellum (Cerebelum_3_L), insula (Supp_Motor_Area_R), and intraorbital superior frontal gyrus (Frontal_Mid_2_R) (Figure 5A blue underline). In the NC group, numerous fibers reached the parahippocampal gyrus (ParaHippocampal_L), cerebellum (Cerebelum_6_R), and fusiform gyrus (Fusiform_R) (Figure 5B red underline). Notably, several connection areas were observed in the MCI group, which were not observed in the NC group, such as the intraorbital superior frontal gyrus (Frontal_Mid_2_R), inferior frontal gyrus of island cap (Frontal_Inf_Tri_R), and olfactory cortex (Olfactory_L) (Figure 5A orange underline). Compared with the NC group, the number and range of fibers reaching the parahippocampal gyrus in the MCI group were significantly lower (Figures 4, 5).

In the present study, the main functional areas (hippocampus, bilateral medial temporal lobe, and parahippocampal gyrus) that are responsible for olfactory and cognitive functions were weaker in MCI patients, especially the parahippocampal gyrus and bilateral medial temporal lobe (Figures 4, 5).

### Correlations Between Behavioral and Imaging Indicators

The mean FAs of the orbitofrontal gyrus, hippocampus, inferior occipital gyrus, and parahippocampal gyrus were extracted from the ROIs of all subjects, and Pearson correlation analyses were performed between the MoCA, OI scores and the FA values. Compared with the NC group, the OI score was significantly correlated with the FA value of the orbitofrontal gyrus in the MCI group, followed by the inferior occipital gyrus, and there were significant differences (*p* < 0.05; Table 5). However, in our cohort of MCI group, there were no significant differences between the MoCA score and the FA values.

**TABLE 5:** Correlation between behavioral indicators and FA values of the ROIs.

	**Orbitofrontal gyrus**		**Hippocampus**		**Inferior occipital gyrus**		**Parahippocampal gyrus**	
	***p***	***r***	***p***	***r***	***p***	***r***	***p***	***r***
OI score	<0.001	0.5360	0.049	0.2920	0.001	0.4470	0.415	−0.123
MoCA score	0.050	0.2910	0.720	0.0540	0.101	0.2450	0.134	−0.273

## Discussion

In line with our *a priori* hypothesis, we found that the olfactory ability of MCI patients was impaired overall and was positively correlated with the MoCA score. Using receiver operating characteristic (ROC) analysis, we revealed that the olfactory test value had high specificity for predicting MCI. Impaired olfactory function was associated with lower general cognitive performance, which was associated with a higher prevalence of MCI. This result suggests that OI is an important marker of cerebral neuropathological changes (Devanand et al., 2015; Palta et al., 2018). Indeed, a meta-analysis reported that olfactory function is vulnerable to pathological changes in patients with AD and MCI and that olfactory function is impaired in patients with AD more profoundly than in those with MCI (Jung et al., 2019), which indicates that OI may also be a marker of MCI-to-AD transition risk (Wu et al., 2019).

It is well established that the orbitofrontal gyrus serves as the higher olfactory center. The corpus callosum, which serves as a communication channel between the bilateral cerebral hemispheres, connects the frontal lobes of the bilateral cerebral hemispheres. The frontal lobe is the higher brain area and is responsible for a variety of activities, which include smell and emotion. This explains why abnormalities in the structure of the corpus callosum cause damage to nerve pathways projecting to the frontal lobe, which result in changes in sense of smell and episodic memory. Therefore, structural abnormalities of the corpus callosum should also be regarded as a crucial factor that causes olfactory function impairment. Several studies have confirmed that early pathological changes in AD occur in the primary olfactory cortex (Conti et al., 2013; Roberts et al., 2016), which is consistent with our study. Our TBSS results showed that the areas of disruption in the MCI group were located primarily in the corpus callosum, orbitofrontal gyrus, and left occipital lobe. However, the area around the hippocampus was not significantly different between the groups; moreover, there were fewer fibrous connections between brain regions related to olfaction, memory, and cognition. These findings suggested that, in MCI patients, the microstructure of the orbitofrontal gyrus and corpus callosum was damaged, whereas changes in hippocampal microstructure were not significant. This indicated that white matter damage in regions underlying olfactory function was obvious in MCI patients, whereas white matter damage in areas involved in memory and cognition was not apparent. Taken together, these findings suggest that decline in olfactory function occurs earlier than does cognitive function impairment. The olfactory system was highly connected with entorhinal–hippocampal–cortical and amygdala–parasympathetic clusters, which is in line with previous literature (Ubeda-Bañon et al., 2020). Furthermore, results from a pilot study suggested that the reduction in the size of the hippocampus is associated with a loss of OI ability, rather than the loss of memory in relation to early AD (Kjelvik et al., 2014). However, another study reported that olfactory impairment was associated with white matter lesions that were independent of hippocampal atrophy (Heinrich et al., 2018). Our TBSS results of the MCI group indicated that there were no significant alterations in the area around the hippocampus, which is involved in cognitive and memory functions; thus, indicating that changes in hippocampal microstructure in MCI patients were not significant. This further confirms that impairment in olfactory function is superior to memory deficits for predicting cognitive decline.

As shown in a previous investigation, olfactory changes can appear earlier than typical dementia symptoms and inflict greater cognitive impairment (Josefsson et al., 2017); however, the underlying mechanism and pathology remain unclear. Therefore, we speculate that olfaction impairment is a foremost outcome of the pathological changes in these areas, which suggests that changes in olfactory ability lead to pathological changes in these areas, which cause alterations in cognitive function. In addition, our findings indicated that fibrous connections in several brain regions, such as the entorhinal cortex, were higher in patients with MCI, which is suggestive of a compensatory mechanism of the olfactory pathway. Thus, we speculate that olfactory training may be useful for improving cognition and may contribute to the prevention of various neurodegenerative diseases. In our traditional perception, the olfactory decline observed in aging may seem irreversible, but emerging evidence suggested that olfactory function may be trained, and olfactory training even has positive effects on cognitive function (Birte-Antina et al., 2018). Indeed, if olfactory training can lead to cognitive benefits, what are the mechanisms? One study found that olfactory training led to increased thickness not only in key olfactory structures but also in fronto-temporal areas outside of the olfactory cortex (Al Ain et al., 2019). A similar study showed that functional brain activity changes in a fronto-parietal network associated with higher cognitive abilities under odor identification training (Fournel et al., 2017). Above findings suggested that intensive olfactory training can improve olfactory function and that this improvement is associated with changes in the structure of olfactory processing areas of the brain. It indicated that olfactory training may give a positive future for improvement of cognition.

Based on the principle of TBSS (Smith et al., 2006), abnormal areas detected in TBSS analyses are those areas that have a significantly lower FA value, which indicates that nerve fibers in these areas have reduced integrity and a limited ability for water molecules to diffuse in the same direction. In our study, we found that the orbitofrontal gyrus had a significantly lower FA value in MCI patients compared with NCs, which also correlated with the olfactory score. However, our imaging findings revealed no correlation between the MoCA score and FA value. One study reported that the MoCA score positively correlated with the FA value of the corpus callosum (Mascalchi et al., 2019). We speculate that the reason for this discrepancy is the susceptibility of the experimental results to bias due to our small sample size.

Most previous studies of AD and MCI patients assessed whole-brain white matter, which included both superficial and deep white matter. Recently, one study evaluated only the superficial white matter and found that microstructural changes in superficial white matter are related to clinical symptoms of AD (Bigham et al., 2020). Therefore, we suggest that microstructural changes in the hippocampus may be detected by exploring the superficial white matter. However, further research investigating superficial white matter is needed to ascertain the precise mechanisms of OI dysfunction.

This study has several limitations. Firstly, we used a relatively small sample size, which may have prevented the detection of biological associations. Nevertheless, we were still able to reveal significant differences. Studying a larger group of patients will likely allow further details to be uncovered. Secondly, because the study used a cross-sectional design, we could not infer whether the changes were due to progression to AD. Thirdly, we used the MoCA to diagnose MCI, because there is currently no specific diagnostic tool based on pathological or molecular imaging assessments. However, the MoCA is only to be a screening tool, it is inaccurate especially in cases who refuse to answer survey questions of the MoCA. Hence, in this community cohort, the predictive accuracy of the MoCA for cognitive decline was moderate, which suggested that the MoCA may need to be combined with other measures to improve predictive power.

Studies on MCI are still limited for preventing progression to dementia. The most valuable finding of our study is that the decline in olfactory function may occur earlier than impairment of cognitive function, which suggests that OI is a significant and useful indicator of neuropathological changes and an effective marker for the development of cognitive decline and dementia.

## Conclusion

In summary, the impairment of olfactory function was superior to memory deficits for predicting cognitive decline in cognitively intact participants. We revealed that olfactory function tests are a useful screening tool for cognitive decline in older adults. Moreover, this tool can be used to screen for cognitive decline before the onset of other clinical symptoms of dementia, which will help to reduce delayed and underdiagnoses of MCI and dementia. Taken together, more attention should be given to those with olfactory disorders, because of the associated higher risk of cognitive decline.

## Permission to Reuse and Copyright

We declare that all figures, tables, and images will be published under a Creative Commons CC-BY license. The raw data supporting the conclusions of this article will be made available by the authors, without undue reservation.

## Data Availability Statement

The original contributions presented in the study are included in the article/supplementary material, further inquiries can be directed to the corresponding author/s.

## Ethics Statement

The studies involving human participants were reviewed and approved by the Ethics Review Committee of Shanghai East Hospital. The patients/participants provided their written informed consent to participate in this study. Written informed consent was obtained from the individual(s) for the publication of any potentially identifiable images or data included in this article.

## Author Contributions

YS, BJ, HQ, SH, and YZ helped to collect the behavioral and imaging data. YS wrote the first draft of the manuscript. ZW and BJ helped in MRI data analysis. YZ, QX and GL supervised the initial drafting, and critically revised the manuscript. QX analyzed the article and critically revised the manuscript. All authors read and approved the final manuscript and contributed to the article conception.

## Conflict of Interest

The authors declare that the research was conducted in the absence of any commercial or financial relationships that could be construed as a potential conflict of interest.

## Publisher’s Note

All claims expressed in this article are solely those of the authors and do not necessarily represent those of their affiliated organizations, or those of the publisher, the editors and the reviewers. Any product that may be evaluated in this article, or claim that may be made by its manufacturer, is not guaranteed or endorsed by the publisher.

## Footnotes

1
https://fsl.fmrib.ox.ac.uk/fsl/fslwiki
